# Heat Shock Protein 70 as a Double Agent Acting Inside and Outside the Cell: Insights into Autoimmunity

**DOI:** 10.3390/ijms21155298

**Published:** 2020-07-26

**Authors:** Stefan Tukaj

**Affiliations:** Department of Molecular Biology, Faculty of Biology, University of Gdańsk, Wita Stwosza 59, 80-308 Gdańsk, Poland; stefan.tukaj@biol.ug.edu.pl; Tel.: +48-58-523-6119

**Keywords:** Heat shock proteins, Hsp, Hsp70, autoimmune diseases, inflammation

## Abstract

Heat shock proteins (Hsp) are a diverse group of constitutive and/or stress-induced molecules that are categorized into several classes on the basis of their molecular weight. Mammalian Hsp have been mostly regarded as intracellular chaperones that mediate a range of essential cellular functions, including proper folding of newly synthesized polypeptides, refolding of denatured proteins, protein transport, and stabilization of native proteins’ structures. The well-characterized and highly evolutionarily conserved, stress-inducible 70-kDa heat shock protein (Hsp70), is a key molecular chaperone that is overexpressed in the cell in response to stress of various origin. Hsp70 exhibits an immunosuppressive activity via, e.g., downregulation of the nuclear factor-kappa B (NF-κB) activation, and pharmacological induction of Hsp70 can ameliorate the autoimmune arthritis development in animal models. Moreover, Hsp70 might be passively or actively released from the necrotic or stressed cells, respectively. Highly immunogenic extracellular Hsp70 has been reported to impact both the innate and adaptive immune responses, and to be implicated in the autoimmune reaction. In addition, preclinical studies revealed that immunization with highly conserved Hsp70 peptides could be regarded as a potential treatment target for autoimmune arthritis, such as the rheumatoid arthritis, via induction of antigen-specific regulatory T helper cells (also called Treg). Here, a dual role of the intra- and extracellular Hsp70 is presented in the context of the autoimmune reaction.

## 1. Introduction

Heat shock proteins (Hsp) are a group of constitutive and/or stress-induced molecular chaperones that are categorized into several classes, including Hsp110, Hsp90, Hsp70, Hsp60, Hsp40, and the so-called small Hsp (sHsps) [1]. They assist in proper polypeptide folding, refolding of denatured proteins, protein transport, and stabilize structures of native (physiologically active) proteins [2]. Basic studies and clinical data have indicated that numerous Hsp are overexpressed in inflamed tissues [3,4,5]. Whether overexpression of Hsp in chronically inflamed tissues (typically found in autoimmune diseases) regulates or participates in the pathology process of a given disease remains unclear. On one hand, pharmacological inhibition of the Hsp90 chaperone activity has led to attenuation of the immune response and amelioration of the autoimmune and inflammatory diseases in several animal models [6]. On the other hand, pharmacological co-inducers of Hsp70 expression have down regulated the inflammation process in preclinical models of rheumatoid arthritis (RA) [7,8,9]. The well-characterized and highly evolutionarily conserved stress-inducible 70-kDa heat shock protein (Hsp70) is a key molecular chaperone that is overexpressed in the cell under stress conditions. Hsp70 can be released to the extracellular milieu both, under physiological and stress conditions. There, it acquires new properties and functions [10,11]. Numerous contradictory results suggested that both, the intra- and extracellular Hsp70 can exert a dual role in the autoimmune-like diseases. It seems that such an equivocal role of this chaperone depends on its origin (bacterial or self), site of inflammation, type of the disease [6,11,12,13,14,15], and possibly other undefined reasons. For instance, it has been found that the Hsp70-derived epitopes interact with the immune cell components, consequently stimulating the humoral autoimmune response and secretion of the anti-Hsp70 autoantibodies. Although the anti-Hsp70 autoantibodies are found to be elevated in patients suffering from various autoimmune disorders, their pathological role and value for predicting development of autoimmunity is not fully understood [16,17,18]. In contrast, preclinical studies have shown that immunization with Hsp70 suppresses inflammation in rodent models of autoimmune arthritis [14,19,20,21,22]. This review article presents the physiological role of Hsp70 in the cell and extracellular environment in the context of inflammation and the autoimmune reaction.

## 2. Hsp70: Structure, Expression, and Mechanism of Action

The human Hsp70 (HSPA) family consists of thirteen gene products that differ from each other by expression pattern (constitutive and inducible forms of Hsp70), subcellular location (e.g., cytosol/nucleus, ER, peroxisome, or mitochondria), and amino acid constitution. Stress-induced HSPA1 (collectively referred to as “inducible Hsp70”) is the most studied in this family. The Hsp70 family is characterized by possession of two functional domains, i.e., (i) a 45 kDa N-terminal nucleotide binding domain (NBD) which binds and hydrolyzes ATP, and (ii) a 25 kDa C-terminal substrate binding domain (SBD) which binds to the polypeptide substrates. The NBD and SBD domains are connected by a short flexible linker. While the highly conserved NBD domain is composed of four subdomains surrounding the ATP-binding pocket, SBD is composed of a β-sandwich and an α-helical “lid” domain. The structure of Hsp70 changes and depends on the ATP-binding state. Thus, ATP hydrolysis by Hsp70 is thought to be a major determinant of this chaperone’s function [2,23].

**Figure 1:**
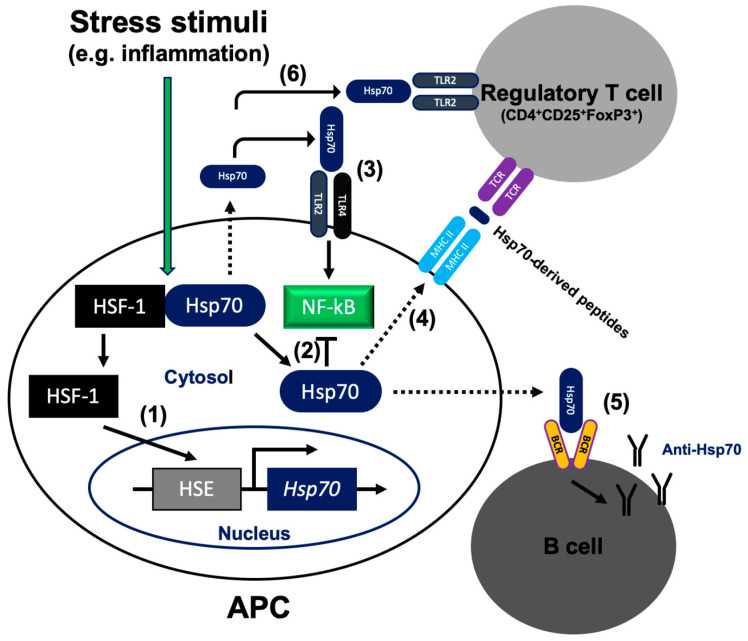
Mode of 70-kDa heat shock protein (Hsp70) action inside and outside the cell. (1) During unstressed conditions, heat shock factor (HSF)1 exists as a monomer in complex with cytoplasmic Hsp70. Upon cellular stress (e.g., inflammation), HSF1 accumulates in the nucleus and binds to the heat shock element (HSE) that is found in the upstream regulatory regions of the Hsp70 gene. (2) Intracellular Hsp70 blocks NF-κB activation. (3) Acting as damage-associated molecular pattern (DAMP), extracellular Hsp70 interacts with TLR2/4 and activates the inflammatory pathway via NF-κB in antigen presenting cells (APC). (4) Extracellular Hsp70 leads to Treg expansion. Hsp70-derived peptides are delivered to the major histocompatibility complex (MHC) class II molecules and presented on the cell surface for recognition by Treg. (5) Extracellular Hsp70 stimulates the humoral autoimmune response and production of the anti-Hsp70 autoantibodies. (6) Extracellular Hsp70 enhances immunosuppressive function of Treg cells via the TLR2 signaling pathway. Dotted lines illustrate translocation of Hsp70 across the cell membrane.

Expression of Hsp is regulated at the transcriptional level by the so-called heat shock factors (HSFs). Among several members of the HSF family (HSF1−HSF4), HSF1 is the best studied [24]. It is an evolutionarily conserved molecule that coordinates stress-induced transcription and directs broad physiological processes in eukaryotic cells. HSF1 is the key component of the heat shock response (HSR), which regulates basal and stress-induced expression of Hsp, including Hsp70 and other targets. In fact, Hsp70 can directly inhibit HSF1, thereby providing a regulatory mechanism that senses polypeptide folding in the cytoplasm and adjusts the extent of stress responses [25,26,27]. Under unstressed conditions, HSF1 exists as a monomer in complex with cytoplasmic chaperones, such as Hsp70 and Hsp90. Upon cellular stress, HSF1 undergoes multiple modifications and forms a transcriptionally active trimer that is translocated to the nucleus where it binds to heat shock elements (HSEs) that are found in the Hsp70 gene’s upstream regulatory regions (Figure 1; pathway no. 1) [28].

It is well established that the three-dimensional structure of a protein is determined by its amino acid (aa) sequence, but the crowded cytoplasmic space often presents obstacles to successful and correct protein folding. Although many polypeptides fold on their own due to physical interactions of their amino acid residues, others require assistance of molecular chaperones to reach their native (physiological) state. The Hsp70 family of molecular chaperones (together with their co-chaperones) assists in a wide range of folding processes, such as folding and assembly of newly synthesized polypeptides, as well as refolding of misfolded and aggregated proteins. The activities mentioned above appear to be based on the Hsp70 property allowing it to interact with exposed hydrophobic peptide residues of proteins in an ATP-controlled fashion [29,30]. In fact, Hsp70 preferentially binds hydrophobic regions of newly synthesized linear peptides or exposed regions of partially unfolded proteins. It seems that such interactions lack strong sequence specificity and allow Hsp70 to bind to a variety of “client proteins”, including cell signaling molecules. For these chaperones, ATP hydrolysis results from binding of substrates to the SBD domain with concomitant NBD interactions with the J-domain of Hsp40 (a co-chaperone). Conformational changes associated with ATP conversion to ADP cause closing of the “lid” and enhance affinity for the substrate. The cycle is completed when Hsp70-specific nucleotide exchange factors (NEF) interact with NBD and assist in ADP release [31].

## 3. Hsp70 Modulates NF-κB Activation

The innate immune cells, including monocytes/macrophages, dendritic cells and granulocytes, are important players in the inflammation process. These cells express pattern recognition receptors (PRRs) on their surface, including toll-like receptors (e.g., TLR2 and TLR4), that detect various microbial components, and the so-called pathogen-associated molecular patterns (PAMPs). In addition, PRRs can recognize molecules that are released by autologous necrotic cells and damaged tissues which are commonly referred to as damage-associated molecular patterns (DAMPs). A common signaling event for PRRs is activation of the nuclear factor-kappa B (NF-κB) pathway [32]. NF-κB was initially discovered as a nuclear factor that binds to the enhancer element of the Ig κ light chain of activated B lymphocytes. Later studies proved that NF-κB in also found in other cell types and regulates various target genes with a whole variety of functions [33,34]. NF-κB is activated in cells by a diverse range of stimuli, such as the TLR, antigen, and cytokine receptors, as well as physical and oxidative stress [35]. It regulates multiple aspects of the innate, but also adaptive immune functions, and controls cell growth [32,36,37]. Therefore, NF-κB is found to play a critical pathological role under inflammatory conditions, including the autoimmune and cancer diseases where its activity is often found to be dysregulated. Both, the intra- and extracellular Hsp70 have been shown to modulate NF-κB activation [38,39,40,41]. Numerous data have proven that intracellular Hsp70 can down regulate the NF-κB activity (Figure 1; pathway no. 2). Mechanistically, overexpressed Hsp70 blocks NF-κB activation and p50/p65 nuclear translocation through inhibition of IKK mediated IκB (NF-κB inhibitor) phosphorylation [39,40,41,42,43,44,45,46]. Interestingly, an opposite effect occurs when Hsp70 is outside the cell. It is speculated that extracellular Hsp70 can act as DAMP via TLR2 and TLR4, and thus can stimulate the immune responses leading to inflammation (Figure 1; pathway no. 3) [47]. Consequently, enhanced expression/secretion of NF-κB-dependent proinflammatory cytokines has been noted in response to extracellular Hsp70 in the human lung cancer cells, dendritic cells, and monocytes, including interleukin (IL)-1β, IL-6, and TNF-α, [48,49,50]. Other studies have found, however, that in synoviocyte cultures obtained from RA patients extracellular Hsp70 inhibits the NF-κB signaling pathway leading to downregulation of IL-6, IL-8, and MCP-1 [51]. In addition, extracellular Hsp70 was shown to negatively regulate production of the pro-inflammatory cytokines, such as TNF-α and IL-6, in monocytes exposed to TLR agonists and contributed to dampening of the inflammatory response [52].

The above-mentioned conflicting results suggest that Hsp70 may play a dual role in the extracellular space that may depend on the type of cells which interact with such chaperones and the type of the disease. It seems that such an equivocal role of Hsp70 also depends on the immunological niche and the general cell culture conditions.

## 4. Hsp70 and Cancer

High expression of Hsp70 allows cells to survive injuries that are lethal under normal conditions and an increased Hsp70 level leads to inhibition of programmed cell death through Hsp70-mediated interactions at several points of the apoptotic signaling pathways. While high expression of intracellular Hsp70 may down-regulate inflammation via the NF-κB signaling pathway, enhanced expression of Hsp70 in cells may be responsible for tumorigenesis and for tumor progression. In addition, such cancer cells can acquire multidrug resistance to chemotherapy [53]. The above-mentioned observations are beyond the scope of this review; however, it is worth mentioning that there are also dark sides to intracellular Hsp70 in the context of cancer development. The majority of human tumors overexpress Hsp70 and upregulation of these chaperones is a typical marker for poor survival and worse prognosis, and is usually correlated with drug resistance, as well as resistance of cancer cells to the immune-mediated destruction [54]. Largely, most anticancer drugs currently used in clinical oncology exploit the apoptotic signaling pathways to trigger cancer cell death. In general, apoptosis is a physiological process that is essential for embryogenesis, development, ageing, and maintenance of cellular homeostasis [55]. In fact, Hsp70 is involved in apoptotic signaling and increases the survivability of cells under stress. While cells with Hsp70 knockdown are sensitive to apoptosis, overexpression of Hsp70 inhibits apoptosis [54]. Colloquially, Hsp70 acts as a “friend” of cancer development due to its anti-apoptotic activity. Hence, researchers are currently attempting to improve cancer treatment therapies by using Hsp70 inhibitors. On the other hand, presence of Hsp70 on the surface of cancer cells may sensitize tumor cells to the cytotoxic attack by natural killer (NK) cells and may elicit a specific anti-tumor response. Therefore, it appears that the intracellular properties of Hsp70 help the cancer cells to develop, while the extracellular Hsp70 may be targeted by the cells of the immune system and thus may help in the development of proper immunotherapy [56,57].

Interestingly, the chaperone Hsp90 has also been linked with the development of many types of cancer. Since Hsp90 is involved in stabilization of multiple oncogenic “client proteins”, its specific chaperone activity inhibitors are currently being tested as anticancer drugs in numerous clinical trials [58]. However, the clinical efficacy of Hsp90 inhibitors, such as geldanamycin (GA) and its derivatives (e.g., 17-DMAG and 17AAG) has been generally disappointing. It is believed that the lack of full success in the treatment of cancer using this type of Hsp90 inhibitors may be due to activation of HSF1 and overexpression of Hsp70 in GA-treated cells, and in this way cancer growth is supported [6]. However, it turned out that these unexpected consequences of using Hsp90 inhibitors belonging to the GA derivatives can be employed in the treatment of inflammatory disorders. Indeed, Hsp90 is involved in activation of the immune response, and pharmacological inhibition of Hsp90 has been successfully used in animal models of inflammatory and autoimmune diseases. Colloquially, it is speculated that the immunosuppressive effects of the anti-Hsp90 therapy are mediated via induction of the Hsp70 expression [6,59,60,61].

## 5. Hsp70 Is Present Outside the Cell

Multiple observations suggested that Hsp70 functions both intra- and extracellularly. In the past, the presence of autologous (self) Hsp in the extracellular space was solely associated with dying (necrotic) cells. However, it had been found by Hightower and Guidon [62] that Hsp, including Hsp70, can be actively released from the cells in the absence of necrosis. Yet, the mechanism of Hsp70 secretion from the mammalian cells is still enigmatic because none of the Hsp70 possess secretory signals. Therefore, it seems that the export of Hsp70 outside of the cell is conducted by different alternative pathways, including the lysosome–endosome pathway [10,11]. However, there is still controversy and conflicting information regarding the role of Hsp70 and other Hsp in the extracellular space in the context of inflammatory diseases. As mentioned above, Hsp70 has been attributed extracellular actions, since it is actively released under inflammatory conditions. Acting as DAMPs, extracellular Hsp70 interacts with the membrane receptors, including TLR2/4 or CD14, and activates the inflammatory pathways [48,49,50,63,64]. However, it is speculated that these proinflammatory properties of Hsp70 may have resulted from the presence of highly immunogenic bacterial endotoxins, such as lipopolysaccharides (LPS), in the recombinant protein preparations produced in bacterial (e.g., *E. coli*) expression systems [11,12]. In contrast to the reported Hsp70 proinflammatory activities, a body of literature indicates that these molecular chaperones can have profound immunosuppressive effects [12,22,65]. It is also hypothesized that the production of recombinant eukaryotic Hsp70 proteins in bacterial expression systems and the absence of post-translational modifications in these proteins (antigens) may lead to equivocal conclusions [66]. Regardless of its extracellular pro-/anti-inflammatory properties, other mechanisms explain the presence of Hsp70 outside the cell and relate to its relationship with the major histocompatibility complex (MHC). In general, antigen presentation includes MHC class I and MHC class II, and is an essential part of the adaptive (acquired) immunity and immunotolerance. While cytotoxic T cells (Tc) specifically recognize MHC class I antigens and eliminate virally infected cells or tumor cells, the T helper cells (Th) classically recognize antigens presented on MHC class II molecules. These latter antigens are presented on the surface of professional antigen presenting cells (APCs), such as the dendritic cells (DC), B cells, monocytes/macrophages, epithelial cells, endothelial cells, and tumor cells [67]. In fact, Hsp70 play a key role in regulation of antigen trafficking and MHC presentation, and self Hsp70 are among the most frequent MHC ligand sources in APCs (Figure 1; pathway no. 4) [21,64,68,69,70,71].

## 6. Extracellular Hsp70 Activates the Humoral Autoimmune Response

Pockley et al. [72] have shown that Hsp70 and anti-Hsp70 antibodies are present in the serum of healthy individuals. It has been found that Hsp70-derived epitopes interact with the immune cell components, consequently stimulating the inter alia humoral autoimmune response and production of the anti-Hsp70 autoantibodies (Figure 1; pathway no. 5). Although the anti-Hsp autoantibodies are found to be elevated in patients suffering from numerous inflammatory and autoimmune diseases, including RA, dermatitis herpetiformis, coeliac disease, and other (auto)inflammatory diseases, their pathological role and value for prediction of the development of autoimmunity is not completely understood [16,17,18,73]. Increased or decreased levels of Hsp70 in the biological fluids (e.g., serum) have been associated with a plethora of clinical conditions that could either act as drivers of pathology or serve as biomarkers of disease and indicators of the disease activity and severity [11]. For instance, higher levels of proinflammatory IL-6 positively correlate with autoantibodies directed towards Hsp40 in RA patients [74]. In addition, positive correlation between the serum levels of Hsp70 and the disease progression and activity in RA may suggest a direct contribution of this chaperone in this disease [75]. However, another study has found a significant inverse correlation for serum levels of anti-Hsp70 autoantibodies (IgM) and proinflammatory TNF-α in RA [18], with no correlation between Hsp70 serum levels and the disease progression and activity in RA [15]. In fact, immunosuppressive effects of antibodies towards microbial- and self-Hsp have been proven in case of Hsp60. For instance, naturally occurring or acquired bacterial (*M. tuberculosis*) anti-Hsp60 antibodies protect against induction of arthritis in the rodent model, such as the adjuvant-induced arthritis (AA) [76]. Pre-treatment of rats with soluble mycobacterial hsp65 (Hsp60) protected against induction of AA that was paralleled by suppression of IL-17, anergy induction, and enhanced serum levels of anti-hsp65 antibodies [77]. In addition, humanized anti-Hsp60 mAb was found to be effective in protecting and suppressing the rodent models of AA or collagen-induced arthritis (CIA), as well as the colitis models [78].

## 7. Hsp70 Promotes Regulatory T Cells

It is well known that the immune system must be able to effectively recognize and fight against microbes while staying tolerant to the body’s own components (autoantigens). This is achieved by the central (a positive and negative selection in the thymus) and peripheral tolerance mechanisms. Regulatory T cells (Tregs) are T helper cells which play a role in regulation or suppression of the other immune system cells. Tregs control the immune response to self and foreign antigens and help prevent the autoimmune reaction. They can be generated in the thymus or can be formed by differentiation of conventional T helper cells in the periphery. Recent studies have identified some (auto)antigens mediating the positive selection of Tregs in the thymus [79]. In fact, contribution of the self Hsp70 in the positive selection of Tregs in the thymus has been already proposed [80]. Therefore, it is tempting to speculate that elevated levels of autologous Hsp70 in circulation (arising in response to stress stimuli, such as inflammation), might also activate the antigen specific Tregs that regulate the adaptive arm of the immune response and protect against its chronic manifestation/feature [19,22,81]. Numerous pre-clinical observations have proven that immunization of animals with bacterial Hsp70 and its highly conserved peptides could be regarded as a potential treatment target for RA and possibly other autoimmune diseases via induction of antigen specific peripheral Treg cells. This experimental therapy ameliorated arthritis development in both, the prophylactic and therapeutic approaches [14,19,20,21,82,83]. Another study showed that Hsp70 enhances the immunosuppressive activity of Treg cells via the PI3K/AKT, JNK, and p38 MAPK pathways, and increases secretion of immunosuppressive cytokines, such as IL-10 and TGF-β in vitro [84]. It is suggested that effects of Hsp70 on human Tregs are dependent on the TLR2 signaling (Figure 1; pathway no. 6) [84]. It is also suggested that the immunosuppressive effects of extracellular Hsp70 on the adaptive arm of the immune response are mediated by induction of tolerogenic dendritic cells [85]. For instance, extracellular Hsp70 has been noted to induce a tolerogenic phenotype in a monocyte-derived DC and inhibited activated T cell proliferation [86]. In addition, Hsp70-specific T helper-cells have been detected in the majority of RA patients and could be converted into a type 1 regulatory (Tr1) cells by tolerogenic DC for a therapy purpose [87]. In contrast, our recent studies have found that elevated Hsp70 serum levels can exert a dual role in RA. While the Hsp70 treatment increased the pro-inflammatory Th17 frequencies and the Th17:Treg ratio, the frequency of pro-inflammatory Th1 cells and the Th1:Th2 ratio were significantly decreased in the Hsp70-treated human PBMC cultures. We postulate that the major Hsp70-mediated immunomodulation contribution includes IL-6 influence on Th17:Treg and Th1:Th2, since expression of this pro-inflammatory cytokine was enhanced by extracellular Hsp70 [15].

In addition to evidence regarding the effect of extracellular Hsp70 on disease modifying Tregs’ generation in vivo, it seems that the pharmacological induction of intracellular Hsp70 also has the ability to promote this immunosuppressive cell fraction. For instance, carvacrol has a capacity to co-induce intracellular Hsp70 expression both in vitro and in vivo. Administration of carvacrol to mice has increased the number of Tregs in their spleen and joints, and almost completely suppressed experimental arthritis [7,8,9].

Interestingly, (auto)immune response can be modulated by using DNA vaccines that encode specific antigens. It has been found that vaccination of animals with plasmids encoding *Hsp70* has suppressed the rodent model of lupus erythematosus or AA [88,89]. This therapy has led to expansion of Treg or shifted T cell response from a proinflammatory Th1 to a regulatory Th2/3 phenotype, respectively [88,89].

## 8. Clinical Perspectives

While therapies using pharmacological co-inducers of Hsp70 or immunization using Hsp70 peptides are promising, the above-mentioned observations are based on preclinical studies using cell cultures or animal models of arthritis with limited data on other autoimmune-like diseases and clinical evidences. In contrast, advanced and promising clinical observations in regard to the other Hsp classes (such as Hsp40 and Hsp60), in patients with RA [90,91,92] or diabetes type I, have been already reported [93,94]. In fact, only a single clinical observation has been provided using an ER Hsp70 family member, BiP, in patients with RA. Twenty-four patients with active RA received a single intravenous infusion of the protein and clinical remission was achieved by patients in the 5 mg and 15 mg groups, but not patients who received 1 mg of BiP or placebo [95]. In addition, the BiP responding RA patients had significantly lower serum concentrations of CRP, VEGF, and IL-8. The authors of that study declared that use of BiP is safe in patients with active RA and leads to clinical improvement in some patients [95].

## 9. Conclusions

The Hsp70 family of molecular chaperones displays various biological functions both, inside and outside the cell. Their intracellular roles do not only involve folding or transport of polypeptides, but they also act as important cell signaling molecules involved in either control of the immune responses or promotion of cancer development. Recent studies have shed light on the role of inducible Hsp70 in extracellular space. Although data on the immunosuppressive properties of intracellular Hsp70 are mostly consistent, their role outside the cell is still not entirely clear due to conflicting reports that are largely based on observations using cell cultures. In addition, conflicting serological observations, regarding the levels of Hsp70 and anti-Hsp70 autoantibodies and their potential contribution to the development or maintenance of the autoimmune diseases, do not present an unequivocal picture concerning the role of Hsp70 in the extracellular space. Finally, there is some evidence that Hsp70 could be considered as a vaccine used to suppress the autoimmune process via induction of regulatory T cells. Despite promising preclinical observations in animal models of autoimmune arthritis, further observations including clinical studies are necessary to address the above-mentioned issues.

## Abbreviations

17-DMAG	17-Dimethylaminoethylamino-17-demethoxygeldanamycin
17AAG	17-N-allylamino-17-demethoxygeldanamycin
ADP	Adenosine diphosphate
AA	Adjuvant-induced arthritis
AKT	Protein Kinase B
APC	Antigen-presenting cell
ATP	Adenosine triphosphate
BCR	B cell receptor
BiP	Binding immunoglobulin protein
CIA	Collagen-induced arthritis
CRP	C-reactive protein
DAMP	Damage-associated molecular patterns
DC	Dendritic cell
ER	Endoplasmic reticulum
GA	Geldanamycin
HSE	Heat shock element
HSF	Heat shock factor
Hsp	Heat shock protein
Ig	Immunoglobulin
IkB	Inhibitor of nuclear factor kappa B
IKK	I kappa B kinase
IL	Interleukin
JNK	c-Jun N-terminal kinases
kDa	Kilodalton
LPS	Lipopolysaccharides
mAb	Monoclonal antibodies
MCP-1	Monocyte chemoattractant protein 1
MHC	Major histocompatibility complex
NBD	Nucleotide binding domain
NEF	Nucleotide exchange factor
NF-κB	Nuclear Factor-kappa B
NK	Natural killer
p38	Mitogen-activated protein kinase p38
PAMP	Pathogen-associated molecular patterns
PBMC	Human peripheral blood mononuclear cell
PI3K	Phosphoinositide 3-kinases
PRR	Pattern recognition receptor
RA	Rheumatoid arthritis
SBD	Substrate binding domain
Tc	Cytotoxic T cell
TCR	T cell receptor
TGF-β	Transforming growth factor β
Th	T helper cell
TLR	Toll-like receptors
TNF-α	Tumor necrosis factor α
Treg	Regulatory T helper cell
VEGF	Vascular endothelial growth factor
